# Nobiletin-loaded composite penetration enhancer vesicles restore the normal miRNA expression and the chief defence antioxidant levels in skin cancer

**DOI:** 10.1038/s41598-021-99756-1

**Published:** 2021-10-12

**Authors:** Mahitab Bayoumi, Mona G. Arafa, Maha Nasr, Omaima A. Sammour

**Affiliations:** 1 Department of Pharmaceutics and Pharmaceutical Technology, Faculty of Pharmacy, The British University in Egypt, Cairo, 11837 Egypt; 2 Chemotherapeutic Unit, Mansoura University Hospitals, Mansoura, 35516 Egypt; 3 Department of Pharmaceutics and Industrial Pharmacy, Faculty of Pharmacy, Ain Shams University, African Organization Unity Street, Cairo, 11561 Egypt

**Keywords:** Skin cancer, Drug delivery, Nanotechnology in cancer

## Abstract

Skin cancer is one of the most dangerous diseases, leading to massive losses and high death rates worldwide. Topical delivery of nutraceuticals is considered a suitable approach for efficient and safe treatment of skin cancer. Nobiletin; a flavone occurring in citrus fruits has been reported to inhibit proliferation of carcinogenesis since 1990s, is a promising candidate in this regard. Nobiletin was loaded in various vesicular systems to improve its cytotoxicity against skin cancer. Vesicles were prepared using the thin film hydration method, and characterized for particle size, zeta potential, entrapment efficiency, TEM, *ex-vivo* skin deposition and physical stability. Nobiletin-loaded composite penetration enhancer vesicles (PEVs) and composite transfersomes exhibited particle size 126.70 ± 11.80 nm, 110.10 ± 0.90 nm, zeta potential + 6.10 ± 0.40 mV, + 9.80 ± 2.60 mV, entrapment efficiency 93.50% ± 3.60, 95.60% ± 1.50 and total skin deposition 95.30% ± 3.40, 100.00% ± 2.80, respectively. These formulations were selected for cytotoxicity study on epidermoid carcinoma cell line (A431). Nobiletin-loaded composite PEVs displayed the lowest IC_50_ value, thus was selected for the in vivo study, where it restored skin condition in DMBA induced skin carcinogenesis mice, as delineated by histological and immuno-histochemical analysis, biochemical assessment of skin oxidative stress biomarkers, in addition to miRNA21 and miRNA29A. The outcomes confirmed that nobiletin- loaded composite PEVs is an efficient delivery system combating skin cancer.

## Introduction

Skin cancer has one of the highest incidence rates compared to other types of cancer, and this rate is currently increasing worldwide^1^, owing to ozone depletion and the accompanying increase in the ultraviolet (UV) radiation reaching the Earth. It is rising at a rate of one million new cases reported annually, with the white population being the chief candidates. The UV radiation is primarily implicated as one of the causes of skin cancer, since it is responsible for the generation of free radicals which develop an oxidative stress when their formation exceeds the skin’s antioxidant defense ability^2^. Therefore, triggering a skin repair process based on delivering antioxidants to the skin could be a very promising strategy for treating skin cancer. Moreover, dysregulation of miRNA expression either oncogenic miRNAs or tumor suppressor miRNAs has been demonstrated in most tumor types including skin cancer^3^. Therefore, studying the regulation of miRNA expression either by up-regulating the tumor suppressor ones or down-regulating the oncogenic ones is another valid strategy to ensure the proper treatment of skin cancer.

The conventional treatment strategies for skin cancer include surgery, photodynamic therapy, radiation as well as chemotherapy and immunotherapy, which are toxic, costly and in some cases ineffective^4,5^. Medicinal plants for treatment of various diseases have shown promise owing to their availability, efficacy and low cost^6,7^. Accordingly, several studies reported the use of nutraceuticals for treatment of skin cancer^6,8–10^, especially if delivered through the topical route of administration which is non-invasive, achieves drug localization, decreases the drug concentration required to obtain the therapeutic effect, and allows for better patient compliance due to ease of self-administration^9^.

Citrus flavonoids possess several pharmacological properties, including antioxidant, anti-inflammatory, and anti-carcinogenic activities^11^. Nobiletin is a citrus flavonoid, which has been reported to inhibit the proliferation of cancer cells, both in vitro and in vivo^12–14^. It was reported that topical delivery of nobiletin inhibited skin tumors multiplication via several mechanisms, such as inhibition of both nitric oxide and superoxide generation, suppression of cyclooxygenase 2 and inducible nitric oxide synthase proteins expression, and inhibition of prostaglandin E2 release^15^. Other beneficial topical effects of nobiletin in skin include anti-oxidant, anti-inflammatory, antimicrobial, anti-aging and anti-acne effects^11,15–18^. Unfortunately, owing to the poor water solubility of nobiletin caused by methoxylation of all its hydroxyl groups, it has low bioavailability, and consequently, limited practical uses^19,20^. Nobiletin has only been topically formulated in a hydrogel delivery system till current date^21^, thus, the encapsulation of nobiletin in nanoparticles intended to deposit in skin layers is expected to enhance its therapeutic outcome by overcoming its poor solubility and aiding its skin permeation. The use of nanotechnology has been explored by researchers to overcome the challenges of conventional topical delivery of natural molecules^22^. Nanoparticles can modify the penetration of the encapsulated substances by increasing their solubility, shielding them against instability causes, and providing their controlled release^23–28^. It has been suggested by researchers that nanoparticles favorably utilize the hair follicle and intercellular routes of penetration^29^, however, all permeation mechanisms are possible.

Vesicular systems such as liposomes, transfersomes, ethosomes and penetration enhancer-containing vesicles are the most frequently utilized systems to accomplish such purpose^30^. Liposomes are considered the older generation of vesicles, after which other modified vesicular systems, containing edge activators in the case of transfersomes, ethanol in case of ethosomes, penetration enhancers in case of penetration enhancer vesicles (PEVs), or the lipid ceramide in case of cerosomes have been introduced. These modifications were primarily introduced to augment the skin deposition/permeation of the drugs encapsulated within the vesicles, since liposomes lack the deep-skin penetration ability, but rather accumulate in the stratum corneum (SC), leading to inefficient drug delivery^31–33^. Moreover, another category of vesicular systems was introduced; composite vesicles, which refer to vesicles composed of more than one material of different properties, mainly obtained by introduction of a polymeric material within the structure of the vesicles^34–36^.

Therefore in the current study, nobiletin as a promising nutraceutical in treatment of skin cancer was loaded in different types of vesicles and composite vesicles. The prepared systems were characterized regarding their physicochemical properties, ex vivo skin deposition, and anti- skin cancer activity in human epidermoid cancer cells A431. The selected formulation was tested for its in vivo performance in skin cancer induced animal model, by histological and immunohistochemical examination, biochemical assessment of oxidative stress biomarkers, and gene expression of two widely studied miRNAs in skin cancer; namely miRNA 21 as an example of oncogenic miRNA and miRNA 29A as an example of tumor suppressor miRNA. To authors’ knowledge, no papers were reported on the formulation of nobiletin nanoparticles for topical administration in any indication till current date. In addition, this study is the first attempt to evaluate the effect of nobiletin on antioxidant enzymes and mi-RNAs in the treatment of skin cancer.

## Materials and methods

### Materials

Nobiletin was purchased from Salus Nutra Inc., China. Soybean phosphatidylcholine (Epikuron 200) was kindly provided as gift by Cargill Co., Germany. Transcutol P was kindly provided as gift by Gattefosse’ Co., France. Sodium acetate was purchased from El-Nasr Pharmaceutical Co., Cairo, Egypt. Methanol HPLC analytical grade and sodium phosphate monobasic were purchased from Fisher Scientific Co., UK. Tween 80, chitosan (M.wt 10,000–300,000 Dalton) and sodium phosphate dibasic 98% extra pure anhydrous were purchased from ACROS organics, Belgium. Chloroform was purchased from POCH, Poland. Glacial acetic acid was purchased from Nile Pharmaceutical Co., Egypt. Ceramide VI was kindly provided as gift by Evonik industries AG, Germany. Adhesive tape was purchased from Scotch TM, China. Dimethylsulfoxide (DMSO), 3-(4,5-dimethylthiazol-2-yl)-2,5diphenyltetrazoliumbromide (MTT), 7,12-dimethylbenz[a]-anthracene (DMBA) (≥ 95% pure) were purchased from Sigma-Aldrich, USA. Liquid paraffin and formalin were purchased from El-Gomhoria Pharmaceutical Co., Egypt. Hematoxylin and Eosin were purchased from Merck Chemicals Co., Germany. Anti-Ki67 antibody immunostain was purchased from Abcam Co., Germany (ab 15,580).

### Preparation of nobiletin-loaded vesicular systems

The thin film hydration was the method of choice for the preparation of nobiletin vesicles. Fifty mg of the drug and diverse amounts of soybean phosphatidylcholine (PC) (with or without ceramide), and penetration enhancer (PE) or surfactant as shown in (Table 1) were placed in a glass flask, and dissolved in chloroform: methanol mixture (2:1, v/v)^37,38^. The organic solvent mixture was removed by rotary evaporator under reduced pressure at 40 °C and 120 rpm till a thin film became apparent on the inner surface of the flask. The lipid film was hydrated using either 10 ml phosphate buffer (pH 7.4) to prepare normal vesicles, or 10 ml acetate buffer containing 0.6% chitosan (pH 4.5) to prepare composite vesicles^39^. The vesicles dispersion was mechanically rotated for 30 min at 60 °C, followed by sonication using a probe sonicator (Sonics, VCX130FSJ, USA) for 6 min, and stored at 4 °C until further characterization^37^.

**Table 1:** Composition of different nobiletin loaded vesicular systems.

Formula code*	Composition				Hydrating medium	Type of vesicles
	Lecithin (mg)	Ceramide (mg)	Transcutol†	Tween80†		
F1	200	–	–	–	Phosphate buffer (pH 7.4)	Liposomes
F2		–	2.5	–		PEVs
F3		–	5	–		
F4		–	7.5	–		
F5		–	10	–		
F6		–	–	0.1		Transfersomes
F7		–	–	0.2		
F8		–	–	0.3		
F9		–	–	0.4		
F10		–	2.5	–	Acetate buffer containing 0.6% chitosan (pH 4.5)	Composite PEVs
F11		–	5	–		
F12		–	7.5	–		
F13		–	10	–		
F14		–	–	0.1		Composite transfersomes
F15		–	–	0.2		
F16		–	–	0.3		
F17		–	–	0.4		
F18	200	100	–	–		Composite cerosomes
F19			2.5	–	Phosphate buffer (pH 7.4)	Cerosomal PEVs
F20			5	–		
F21			7.5	–		
F22			10	–		
F23			–	0.1		Cerosomal transfersomes
F24			–	0.2		
F25			–	0.3		
F26			–	0.4		
F27			2.5	–	Acetate buffer containing 0.6% chitosan (pH 4.5)	Composite cerosomal PEVs
F28			5	–		
F29			7.5	–		
F30			10	–		
F31			–	0.1		Composite cerosomal transfersomes
F32			–	0.2		
F33			–	0.3		
F34			–	0.4		

*All formulations contained 50 mg nobiletin.

^†^Added as %w/v of the total volume of the formulation.

### Characterization of nobiletin loaded vesicular systems

#### Particle size, polydispersity and zeta potential measurement

The prepared nobiletin vesicular systems were analyzed for their size, polydispersity indices (PDI) and zeta potential (ZP) using Zetasizer (model Nano-ZS 90, Malvern Instruments, UK) after appropriate dilution using deionized water^40^.

#### Determination of nobiletin entrapment efficiency (EE%) and loading (%DL)

Free nobiletin was separated by centrifugation/filtration using Nanosep centrifuge tube containing an ultra-filter membrane (M.wt cut-off 100 kDa, OMEGA, Pall Corporation, USA). The Nanosep was centrifugated at 3000 rpm for 2 h at room temperature with cooling centrifuge (Centurion Ltd, England)^9^. An aliquot of the filtrate was diluted with HPLC grade methanol, and then the amount of free drug was determined spectrophotometrically at 326 nm (Jasco, V-630, Japan). The entrapment efficiency (EE%) was calculated as a percent of the original amount of drug added according to the following equation:

$${\text{EE}}\% = \, \left[ {{\text{Wt}} - {\text{ Wf }}/{\text{ Wt}}} \right] \, \times \, 100$$

where Wt is the original amount of drug added to the formulations and Wf is the amount of free drug analyzed in the supernatant. The drug loading (%DL) was also calculated as a percent of the amount of encapsulated drug to the weight of the vesicles.

#### Determination of viscosity

Viscosity measurements were conducted for the selected vesicles using Anton Paar rheometer (Anton paar MCR, 302, Australia) connected to plate of 2.5 cm diameter with a fixed shear rate (1/s) at 100 rpm and temperature 25 °C^34,41^.

#### Ultra-performance liquid chromatography (UPLC) analysis of nobiletin for ex vivo deposition/ permeation study

Quantification of nobiletin in the ex vivo deposition/permeation study was carried out using UPLC (Thermo Scientific UPLC-Ultimate 3000, USA) by simple amendment of the method established by other authors^42,43^. The mobile phase was a combination of methanol: water (72:28), flow rate 0.8 ml/min and the injection volume was 20 µl. Samples were injected into a C18 column (Thermo Scientific 2.2 µm, 2.1 × 100 mm) and detection was made at 332 nm.

#### Ex-vivo permeation and skin deposition study

The protocol of the ex-vivo experiment was approved by the Research Ethics Committee of the British University in Egypt (Serial No. EX-2002), and Ain Shams University (REC-MSc. No.112), in accordance with the National Institutes of Health guide for the care and use of laboratory animals (NIH publication No.8023, revised 1978) and ARRIVE guidelines. The ex-vivo permeation and skin deposition study was conducted on the selected vesicular systems using vertical locally fabricated diffusion cell system^44^. Mice were sacrificed by cervical dislocation and the skin was separated, followed by hair removal and washing. The excised skin pieces were placed between the donor and receptor compartments in diffusion cells of area 0.79 cm^2^. The release medium was phosphate buffer saline (pH 7.4) with tween 80 (5.5%) to increase drug solubility and ensure sink condition. Stirring of the medium was performed at 100 rpm at 37 ± 0.2 °C^45^. A constant amount (100 µL) of the selected nobiletin vesicles was placed in the donor compartment. Sequential samples were collected from the receptor compartment at predetermined time intervals (0.5, 1, 2, 4,6,7,8 and 24 h) via the sampling port and assayed by UPLC at 332 nm. After 24 h, the remaining formulation on the skin surface was removed, and tape stripping was performed ten times with adhesive tape to separate the SC^46,47^. The epidermis and dermis were then separated using forceps. Pieces of the adhesive tape as well as epidermal and dermal layers were placed in methanol. Each skin layer was then sonicated for 30 min to extract the deposited drug. Finally, all samples were injected into the UPLC column to determine the deposited percent of nobiletin.

#### Transmission electron microscope (TEM)

The morphological features of the selected nobiletin vesicles were visualized using TEM as previously described^41,48^. Electron micrographs were obtained using JEOL GEM-1010 transmission electron microscope at 70 kV.

#### Physical stability of selected nobiletin vesicular systems

Nobiletin formulations were stored in sealed vials at 4 ± 1 °C for 3 months^9,48,49^. After this period, samples were re-assessed for their particle size, polydispersity indices, zeta potential and entrapment efficiency percentages to assess their stability.

#### *In-vitro* cytotoxicity assay

Human epidermoid carcinoma cell lines (A431 cells) were purchased from ATCC, USA, and treated as previously described^9^.

Cell cytotoxicity was investigated using the MTT assay on human epidermoid carcinoma cell line (A431 cells)^9,50,51^. A stock solution of nobiletin in distilled water and 0.5% dimethyl sulfoxide (DMSO) was prepared as control and diluted using culture media to achieve eight different concentrations (0–500 µg/ml). Nobiletin-loaded composite PEVs (F13) and composite transfersomes (F17) were also similarly diluted to achieve nobiletin concentrations within the same range (0–500 µg/ml), followed by incubation for either 24 or 48 h. After the incubation period, the number of viable cells was evaluated by the MTT assay which is regarded as a sensitive and reliable colorimetric assay to quantify the cellular viability, proliferation and activation^52^. The viability percent was calculated as previously described^53^, and the 50% inhibitory concentration (IC_50_) was calculated from the dose–response curves.

#### *In-vivo* anti- skin cancer activity

The study was carried out on male Balb/C mice (6–7 weeks old) weighing 20 g. Mice were acclimatized for 2 weeks before the experiment. The protocol of the experiment was approved by the Research Ethics Committee of the British University in Egypt (Serial No. EX-2002), and Ain Shams University (REC-MSc. No.112), in accordance with the National Institutes of Health guide for the care and use of laboratory animals (NIH publication No.8023, revised 1978) and ARRIVE guidelines. DMBA was the carcinogen of choice for skin cancer induction^54^. Before performing the experiment, a preliminary study was conducted to decide the dose and frequency of DMBA application required to induce skin cancer. Then, dorsal hair of mice was removed by shaving.

The skin of the dorsal side of the mice was topically applied DMBA (25 µg in 0.1 ml liquid paraffin/mouse) three times weekly over a period of 3 weeks^55^. All mice were observed daily and weighed weekly**.** Only tumors with diameter more than 2 mm and existing for not less than two weeks were recorded^56^. The animals were divided into 4 groups of 15 mice each:

Group I: The negative control group (no cancer induction, no treatment).

Group II: The positive control group (cancer induction, no treatment).

Groups (III–IV) were topically administered nobiletin formulation or solution (0.1 ml/cm^2^) skin area every 24 h.

Group III: received topical application of nobiletin–loaded composite PEVs (F13) (prepared 200 mg lecithin, 50 mg nobiletin, 10% w/v transcutol and hydrated with 10 ml acetate buffer containing 0.6% chitosan) on the hairless dorsal skin every 24 h for 2 months.

Group IV: received topical application of nobiletin solution (dissolved in 5.5% aqueous solution of tween 80) on the hairless dorsal skin every 24 h for 2 months.

Animals were sacrificed after treatment by cervical dislocation, and skin tissue samples were collected and fixed in 10% formalin for examination, followed by treatment, staining and histological evaluation^57^.

For the immunohistochemical study, ki-67 antigen expression in different tissue samples was evaluated using immunohistochemical staining with monoclonal antibody MIB1. Several studies confirmed that ki-67 is one of the best proliferation markers for cancer cells owing to its abundance in all active phases of cell cycle and its absence in resting cells^58,59^. Moreover, ki-67 has been studied as a proliferation marker for skin cancer in particular in several studies^60,61^. One of the most commonly used methods for measuring tumour proliferation is assessment of the fraction of ki-67 positively stained tumour cells. A score was given according to the percentage of positive cells as follows: (0) < 24% positive cells, (+)24–50% isolated positive cells, (+ +) 51–74% focal positive cells, (+ + +) > 75% diffuse positive cells^59^.

For the biochemical study of oxidative stress biomarkers and gene expression in skin, the skin was treated as previously described^9^, and the supernatant of skin homogenates was used to estimate the biochemical parameters; glutathione (GSH), superoxide dismutase (SOD), catalase (CAT) activity levels, malondialdehyde (MDA) as well as miRNA 29A and miRNA 21^9^.

The enzymatic recycling method was used to assess GSH level in the skin^62^ using glutathione Kit (BioVision Co,. USA, CAT NO. K264-100). The concentration of GSH was expressed as µmol/mg tissue protein.

Thiobarbituric acid (TBA) assay is used for measurement of products of lipid peroxidation in animal tissues owing to its simplicity, reproducibility and low cost, thus this assay was implemented in our study for the measurement of MDA level as a secondary degradation product of lipid peroxidation^63^ using malondialdehyde Kit (BioVision Co,. USA, CAT NO. K739-100). The results were expressed as µmol/mg tissue protein^9^.

Superoxide dismutase (SOD) catalyzes the dismutation of the superoxide anion into hydrogen peroxide and molecular oxygen. The sensitive superoxide dismutase kit (BioVision Co. USA CAT NO. K335-100) utilizes WST-1 that is reduced by superoxide anion producing a water-soluble formazan dye; hence, the rate of reduction with a superoxide anion is inversely related to SOD concentration. Therefore, the inhibition activity of SOD can be determined by a colorimetric method^64^. The activity of the enzyme was expressed in U/mg of skin, where one unit (U) is defined as the amount of enzyme that causes inhibition of WST-1 reduction.

The catalase activity was determined using Catalase kit (BioVision Co., USA, CAT NO. K773-100). The activity of the enzyme was expressed in U/mg of skin, where one unit (U) is defined as the amount of enzyme that decomposes 1 μmol of H_2_O_2_ per min.

miRNAs were extracted from 30 mg tissue by miRNeasy extraction kit (Qiagen, Valencia, CA, USA), followed by conversion of individual miRNAs into the corresponding cDNA with a microRNA reverse transcription kit (Applied Biosystems, Massachusetts, USA, CAT NO. 4366597). After the RT-PCR run, the data were expressed in Cycle threshold (Ct) that is the number of qPCR cycles required for the fluorescent signal to cross a specified threshold. The PCR data sheet included Ct values of miRNA-29A and miRNA 21 expressions and the house keeping (reference) gene (the endogenous small nuclear RNA, RNU6B), since gene expression has to be done in comparison with a negative control (reference) sample. Therefore, relative quantitation (RQ) of target gene expression was determined and normalized to reference (internal control) gene^65^.

### Statistical analysis

One-way ANOVA followed by either Tukey or Dunnett’s post-tests were used to perform the statistical analysis of the data using GraphPad InStat 3.1 software.

## Results and discussion

### Preparation of nobiletin vesicles

Nobiletin vesicles were prepared successfully using the thin film hydration method. PC, PE and nobiletin were dissolved in an organic solvent mixture of chloroform and methanol (2:1 v/v) based on preliminary solubility studies conducted on nobiletin, ensuring its solubility in both solvents. The thin film hydration was the method of choice for preparation of vesicles since nobiletin is a hydrophobic drug; therefore this method ensures the production of multilamellar vesicles which increase the entrapment of hydrophobic drugs^38,66^.

As shown in Table 1, two hydrating media were utilized; phosphate buffer saline (pH = 7.4) for liposomes, PEVs and transfersomes and acetate buffer (pH = 4.5) for composite vesicles containing chitosan (0.6%), which were considered acceptable for topical formulations administered to humans^67^. Composite vesicles were prepared in order to investigate whether they can achieve better topical delivery caused by interaction of the positively charged chitosan moiety with the negatively charged surface of the skin^39^.

As shown in Table 1, ceramide was incorporated in some formulations as an additional lipid component, owing to the fact that it is more lipophilic than lecithin, with the ability to create a sufficient space for drug encapsulation and prevention of its leakage^68,69^. Besides, two types of penetration enhancers were utilized in our study; transcutol and tween 80 as they were reported to enhance the skin deposition of lipophilic drugs. Transcutol acts as a skin penetration enhancer which interpenetrates the phospholipid bilayer, thus improves the vesicular bilayer fluidity, and reduces the barrier function of SC^70^. Besides, it is non-toxic and biocompatible, with both aqueous and oil solubility, therefore it was profoundly used in drug delivery^39,71,72^. Tween 80 is a non-ionic surfactant which is also used as penetration enhancer by increasing the fluidity of the lipid content of SC followed by vesicles penetration. It also interacts with keratin fibrils and corneocytes. Moreover, tween 80 destabilizes the vesicular lipid bilayer and increases its deformability by decreasing the interfacial tension^31,73^. The chosen percentages of transcutol and tween 80 concurred with those reported in the literature^74,75^.

### Particle size, polydispersity and zeta potential measurement

As shown in Table 2, the particle size values of nobiletin vesicular formulations (F2-F17) ranged from (69.30–194.50 nm) showing decrease in particle size than their corresponding liposomes (F1) with particle size 616.00 nm, this may be attributed to the presence of edge activator in case of transfersomes or penetration enhancer in case of PEVs which destabilize the lipid bilayer, reducing its surface tension and rigidity, hence, decreasing the vesicular size^76^. The size range of the prepared vesicles perfectly suits drug delivery across the skin, since it was reported by Danaei et al.^77^ that vesicles with diameter 600 nm or above cannot deliver the drug to deep skin layers, in other words they are more likely to deposit on the skin surface or in the stratum corneum, however, vesicles with 300 nm diameter or less can effectively deliver entrapped drug into deeper layers of the skin. Formulation F5 (PEVs) prepared with the highest concentration of transcutol (10% w/v) exhibited the smallest particle size among all vesicle types. Worth mentioning is that the particle size of cerosomes (F18-F34) was not determined owing to the fact that cerosomes are not spherical vesicles but have tubular like morphology as reported by Abdelgawad et al.^34^, consequently PDI and zeta potential were not determined as well. A representative transmission electron microscopy picture showing the tubular morphology of cerosomes is shown in supplementary 1. As apparent in Table 2, PDI values of most formulations (F1–F17) were in the range of (0.3–0.5), thus reflecting homogeneity of the preparations^71,78^ with only few formulations exhibiting a PDI more than 0.5.

**Table 2:** Properties of nobiletin-loaded vesicular systems.

Formula code*	Particle size (nm)	PDI	Zeta potential (mV)	EE%	%DL
F1	616.00 ± 11.00	0.66 ± 0.19	− 29.80 ± 9.60	60.70 ± 2.60	12.14 ± 0.74
F2	134.40 ± 15.50	0.41 ± 0.07	− 14.60 ± 2.00	65.60 ± 4.60	13.12 ± 1.30
F3	123.20 ± 1.30	0.31 ± 0.06	− 8.30 ± 1.00	67.70 ± 7.70	13.54 ± 2.18
F4	115.60 ± 7.10	0.31 ± 0.06	− 5.50 ± 3.00	71.90 ± 6.40	14.38 ± 1.81
F5	69.30 ± 1.90	0.43 ± 0.07	− 9.10 ± 0.80	74.70 ± 1.60	14.94 ± 0.45
F6	128.30 ± 7.20	0.70 ± 0.14	− 12.30 ± 1.20	67.10 ± 5.20	13.41 ± 1.47
F7	113.40 ± 5.60	0.39 ± 0.02	− 10.00 ± 0.50	66.00 ± 3.80	13.19 ± 1.07
F8	111.60 ± 20.60	0.51 ± 0.10	− 14.40 ± 5.70	64.20 ± 10.00	12.83 ± 2.82
F9	74.10 ± 7.50	0.44 ± 0.20	− 14.00 ± 0.20	54.00 ± 6.90	10.78 ± 1.95
F10	194.50 ± 31.10	0.39 ± 0.01	+ 10.20 ± 3.50	89.20 ± 0.80	14.39 ± 0.18
F11	158.90 ± 0.50	0.32 ± 0.05	+ 12.10 ± 1.40	94.70 ± 0.20	15.28 ± 0.05
F12	148.90 ± 15.50	0.41 ± 0.04	+ 10.70 ± 1.20	95.60 ± 2.80	15.42 ± 0.64
F13	126.70 ± 11.80	0.36 ± 0.00	+ 6.10 ± 0.40	93.50 ± 3.60	15.08 ± 0.82
F14	163.70 ± 4.80	0.59 ± 0.01	+ 10.20 ± 0.90	94.90 ± 3.00	15.31 ± 0.69
F15	140.30 ± 10.30	0.57 ± 0.01	+ 12.00 ± 1.40	96.10 ± 1.40	15.49 ± 0.33
F16	122.70 ± 10.70	0.48 ± 0.13	+ 13.90 ± 2.00	95.70 ± 3.30	15.42 ± 0.75
F17	110.10 ± 0.90	0.39 ± 0.01	+ 9.80 ± 2.60	95.60 ± 1.50	15.40 ± 0.34
F18	–	–	–	84.50 ± 2.00	12.08 ± 0.40
F19	–	–	–	77.70 ± 5.30	11.15 ± 1.01
F20	–	–	–	83.10 ± 1.20	11.87 ± 0.24
F21	–	–	–	80.70 ± 1.00	11.53 ± 0.19
F22	–	–	–	76.80 ± 6.00	10.97 ± 1.22
F23	–	–	–	82.60 ± 6.20	11.79 ± 1.25
F24	–	–	–	87.90 ± 0.70	12.55 ± 0.14
F25	–	–	–	86.30 ± 1.50	12.32 ± 0.30
F26	–	–	–	87.60 ± 3.70	12.50 ± 0.75
F27	–	–	–	83.70 ± 4.60	10.21 ± 0.79
F28	–	–	–	80.60 ± 8.30	9.83 ± 1.43
F29	–	–	–	80.20 ± 0.30	9.78 ± 0.06
F30	–	–	–	79.60 ± 6.90	9.71 ± 1.19
F31	–	–	–	94.80 ± 4.40	11.56 ± 0.76
F32	–	–	–	93.60 ± 2.30	11.41 ± 0.39
F33	–	–	–	95.00 ± 1.30	11.58 ± 0.23
F34	–	–	–	95.40 ± 1.30	11.62 ± 0.23

*The particle size of cerosomes (F18–F34) was not determined owing to the fact that cerosomes are not spherical vesicles but have tubular like morphology.

As obvious from the particle size results, the increase in transcutol percentage from 2.5 to 10% showed significant (*P* < 0.05) decrease in particle size in both PEVs and composite PEVs. Such decrease may be attributed to the ability of transcutol to interpenetrate the phospholipid bilayer and modify its packing by lowering its surface tension, consequently leading to smaller particle size^71,76^. In addition, the increase in the percentage of tween 80 from 0.1 to 0.4% led to a significant decrease in the particle size of transfersomes and composite transfersomes (*P* < 0.05) which is attributed to the amphiphilic nature of tween 80, leading to reduction of surface tension, destabilization of the formed vesicles and consequently smaller particle size^73^. On the other hand, formulations containing chitosan showed significantly higher particle size (*P* < 0.05) than their corresponding vesicles prepared without chitosan, which could be ascribed to the interaction of the positively charged chitosan with the negatively charged phospholipids leading to effective coating of the bilayers^39^. This also concurred with the results of other authors who reported an increase in the particle size of their vesicles upon coating with chitosan^79,80^.

Regarding zeta potential results, the negative charge on nobiletin liposomes, PEVs and transfersomes could be ascribed to the presence of fatty acids in lecithin (Epikuron 200)^81^, while the positive charge of composite vesicles is attributed to the presence of chitosan with its protonated amine groups^39,80,82^. The zeta potential values of the nobiletin PEVs ranged from (− 5.5 to − 14 mV). Comparing the zeta potential results of PEVs formulation (F2) and transfersomal formulation (F6) to liposomal formulation (F1), it could be depicted that they were both significantly less negatively charged than liposomes of charge (− 29.8 mV) (*P* < 0.05). These results were similar to those obtained by other authors^75,83^, and could be attributed to the fact that phosphate groups of phosphatidylcholine are oriented towards the external surface of liposomes, however in case of PEVs, interaction between PE and phospholipid changes this orientation leading to decrease in negative charge on the external surface of particles. Increasing the percentage of transcutol from 2.5% (F2, F10) to 10% (F5, F13) significantly decreased the surface charge of both PEVs and composite PEVs (*P* < 0.05) respectively. This came in accordance with Manca et al.^75^, stating that the progressive increase in transcutol percentage in PEVs causes lowering of the dielectric constant of the solvent, leading to partial tilting of the phospholipid hydrophobic tails from the inner part of the membrane to the solvent phase, with overall decrease in the surface charge of the vesicles. The zeta potential values of nobiletin transfersomes ranged from (− 10.04 to − 14.4 mV), which similar to PEVs were significantly less negative than that of liposomes with charge (− 29.8 mV) (*P* < 0.05). These results were similar to what was reported by Zeb et al.^33^ and could be interpreted by the hydrophilic nature of tween 80, causing it to reside on the surface of phospholipid bilayer, hence masking the negative charge of phosphatidylcholine^84^. Worthy to note is that the amount of tween 80 did not significantly affect the zeta potential of either transfersomes or composite transfersomes (*P* > 0.05). The presence of chitosan in the vesicles converted the charge to positive, ranging (+ 6 to + 13.9 mV). The positive charge of chitosan is attributed to the fact that the amino groups of glucosamine residues of chitosan become protonated at acidic pH (pH = 4.5 of our utilized buffer), hence leading to the effective interaction of the positively charged chitosan with the negatively charged phospholipid and bilayer coating^39,80^.

### Entrapment efficiency (EE%) and loading (%DL)

All the prepared vesicular systems exhibited high EE% for nobiletin, which could be ascribed to the lipophilicity of nobiletin (log *P* = 2.48), causing its effective encapsulation within lipid bilayers^39,85^, with %DL ranging from 9.71 to 15.49%. Besides, the thin film hydration method generates large multilamellar vesicles, which results in greater encapsulation of lipophilic drugs in the multiple lamellae^66^. Comparing PEVs formulation (F2) and transfersomes formulation (F6) to liposomes (F1), it can be depicted that the incorporation of either transcutol or tween 80 had insignificant effect on the EE% of nobiletin (*P* > 0.05), similar to results obtained by Manconi et al.^71^ and Mura et al.^72^. Similarly, the incorporation of transcutol (F19) or tween 80 (F23) did not significantly affect the EE% of composite cersomes (F18) (*P* > 0.05).

In case of regular vesicles (F2–F9), the inclusion of ceramide (F19–F26) led to a significant increase in the EE% of nobiletin (*P* < 0.05). The higher EE% in ceramide containing vesicles compared to their counterparts not containing ceramide is attributed to the more lipophilicity of ceramide compared to lecithin, as well as the ability of ceramide to create a matrix for drug encapsulation^34,68^. Ceramide is a very lipophilic molecule belonging to sphingolipids family; its backbone is composed of long chain sphingoid base with another chain composed of amide linked fatty acid^86^, however, ceramides can not form bilayers alone^34,87–89^. On the other hand, in case of composite PEVs (F10-F13), the inclusion of ceramide (F27-F30) displayed a significant decrease in the EE% of composite PEVs (*P* < 0.05). Chitosan was reported to provide a coating layer on phospholipid based membranes, which prevents leakage of drugs^90^, and as previously stated, ceramide increases the lipophilicity of membrane, also increasing the EE% of drugs. Therefore, combined together, the presence of ceramide and chitosan was expected to increase the EE% of nobiletin. However, in the presence of transcutol in composite PEVs, ceramide and chitosan might have displaced part of the transcutol, since transcutol was reported not to be inserted deeply within the phospholipid bilayer, but rather interacting with the phospholipid bilayer while mostly in the aqueous phase^37^. Therefore, the combined presence of ceramide and chitosan might have expelled transcutol totally towards the aqueous phase, taking an additional amount of solubilized nobiletin, consequently increasing the amount of unentrapped nobiletin. Regarding composite transfersomes (F14–F17), a non-significant change in the EE% was observed upon ceramide incorporation (F31–34) (*P* > 0.05), since they are of inherently high EE% values (94.9%-96.14%).

The addition of chitosan showed significant increase in the entrapment efficiency of nobiletin (*P* < 0.05) in both ceramide containing and non-containing vesicles. This complied with the results of Zhou et al.^90^ who attributed these findings to the fact that chitosan either coats the vesicles or embed itself into the bilayer, hence precluding drug leakage from the vesicles. This increase in EE% may also be ascribed to the increase in particle size in composite vesicles, compared to their counterparts not containing chitosan.

The formulations containing the largest amount of transcutol in PEVs and tween 80 in transfersomes exhibiting the smallest particle size (F5, F9, F13, F17), and their counterparts containing ceramide (F22, F26, F30, F34) were selected for further characterization. Formulations (F1 and F18) were also included in the characterization for comparative purposes.

### Characterization of the selected vesicles

#### Determination of viscosity of the selected vesicles

Viscosity of nobiletin vesicular systems was found to be in the range of (1.18–39.40 cP) (Supplementary 2). Obviously, the viscosity of vesicles is attributed to the superior hydrodynamic volume exhibited by the presence of vesicular lamellar structures. The high viscosity of the vesicles favors the adherence of the formulations to the skin and consequently enhanced *in-vivo* skin deposition^37,41^. Comparing PEVs (F5&F22) to their non- penetration enhancer containing counterparts (F1&F18), it was evident that the addition of transcutol did not significantly affect the viscosity of the vesicles (*P* > 0.05). On the other hand, the addition of tween 80 (F9&F26) significantly increased the viscosity of transfersomes (*P* < 0.05) compared their non tween 80 containing counterparts (F1&F18). These results were similar to those obtained by other authors^34^ who reported that tween 80 increases the viscosity of transfersomes owing to its inherent viscosity. The prominent increase in viscosity was observed with the inclusion of chitosan (F5/F13, F9/F17, F22/F30, F26/F34), or ceramide (F1/F18, F5/F22, F9/F26, F13/F30, F17/F34). Chitosan is a well-known viscosity enhancing polymer, hence, formulations containing chitosan exhibited significantly higher viscosity than their corresponding ones without the polymer (*P* < 0.05)^39^. The addition of ceramide as bilayer forming lipid with lecithin led to a significant (*P* < 0.05) increase in the viscosity of cerosomes over non ceramide-containing vesicles, similar to what was reported by other authors^34^, and could be attributed to its solid pasty character^91^.

#### Ex vivo deposition/permeation of nobiletin vesicles

**Figure 1:**
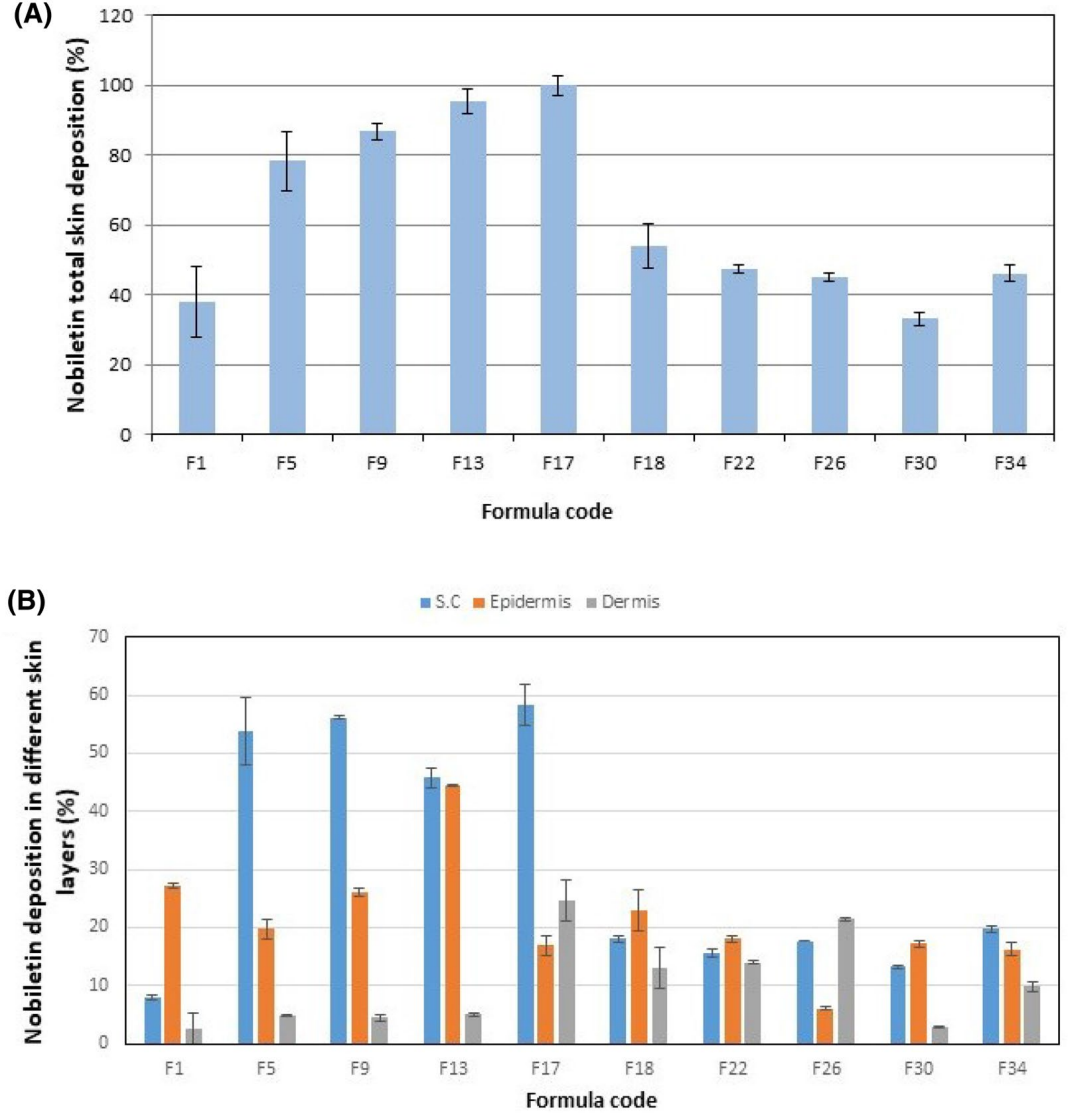
(**A**) Total deposition percent of nobiletin in skin layers (**B**) Deposition percent of nobiletin in S.C, epidermis and dermis.

Ex-vivo deposition/penetration of nobiletin loaded vesicles in mouse skin was investigated, and results are shown in Supplementary 3 and Fig. 1. Since our chief goal in this study was to develop nobiletin vesicular system capable of treating skin cancer by local action, thus we were concerned with nobiletin deposition in different skin layers rather than its permeation. The quantity of nobiletin accumulating in the stratum corneum (SC), epidermis and dermis was expressed as a percentage of the total drug amount applied onto the skin^9,37^.

By inspection of the results, it was obvious that nobiletin in all formulations was successfully deposited in the skin, with total deposition percentages ranged from 33.20–100%. Studying the effect of formulation composition on nobiletin total skin deposition, it was observed that the presence on transcutol (F5) significantly increased the deposition of nobiletin (*P* < 0.05), compared to liposomal formulation (F1). Transcutol PEVs are suggested to penetrate intact to the epidermis, disturbing the densely packed skin lipid pathway and decreasing the barrier function of SC transiently, thus allowing the fluidized vesicles to reach deep skin layers^72,92^. Similarly, the presence of tween 80 (F9) in the transfersomes resulted in a significantly higher total deposition in the skin compared to the liposomal formulation (F1) (*P* < 0.05). Transfersomes, being deformable vesicles are able to squeeze across the tiny hydrophilic intercellular pores of the skin that can not be penetrated by other non deformable lipid aggregates, and the driving force for such mechanism is the osmotic gradient across the skin resulting from the difference in water content between the superficial skin layers, allowing transfersomes to pass spontaneously across the skin. Ceramide caused a significant reduction of the total nobiletin skin deposition (*P* < 0.05). This could be ascribed to the increased affinity of nobiletin to the ceramide, being of higher lipophilicity compared to vesicles prepared using lecithin only, which hinders its release from the cerosomal matrix as free drug^68^. Moreover, the ceramide increases the rigidity of the vesicles, hence hindering the fluidization effect of transcutol or tween 80^34,88^ which explains the non-significant deposition (*P* > 0.05) between the composite cerosomal vesicles (F18), and its counterpart containing transcutol (F22) or tween 80 (F26). The presence of chitosan in non-ceramide containing vesicles (F5/F13, F9/F17) significantly enhanced the total skin deposition of nobiletin (*P* < 0.05), owing to its strong adherence to the negatively charged skin^93^. However, this did not occur with ceramide containing vesicles, in which chitosan addition led to a significant decrease in the deposition of nobiletin in cerosomal PEVs (F22/F30) (*P* < 0.05), and insignificant change in cerosomal transfersomes (F26/F34) (*P* > 0.05), which suggests that ceramide hindered the membrane binding ability of chitosan. This came in contrast to what was encountered with Abdelgawad et al.^34^, in which the structural similarity of ceramide to the natural skin lipids of the stratum corneum resulted in increased deposition of tazarotene in the skin, but came in accordance with El-Zaafarany et al.^94^, who reported the decreased deposition of CoQ10 in cerosomes compared to other vesicular systems. This implies that the physiochemical properties of the encapsulated drugs also play a role in their skin deposition.

Regarding the amount of nobiletin reaching the receptor compartment, it can be observed that almost all vesicles not modified with chitosan displayed some permeated drug in the receptor compartment, while composite vesicles containing chitosan displayed no permeated drug. This could be ascribed to the binding of the positively charged chitosan containing vesicles to the negatively charged skin, leading to better deposition in the skin layers rather than permeation across them.

Based on the aforementioned results, the composite PEVs formulation (F13) and the composite transfersomal formulation (F17) exhibiting the highest nobiletin total deposition (95.30% & 100.00% respectively) were selected for further studies. Worthy to note is that formulations F13 and F17 also exhibited the highest epidermal and dermal deposition respectively among their counterparts, which further delineates their suitability for treatment of basal cell carcinoma (BCC).

#### Transmission electron microscopy (TEM) examination

**Figure 2:**
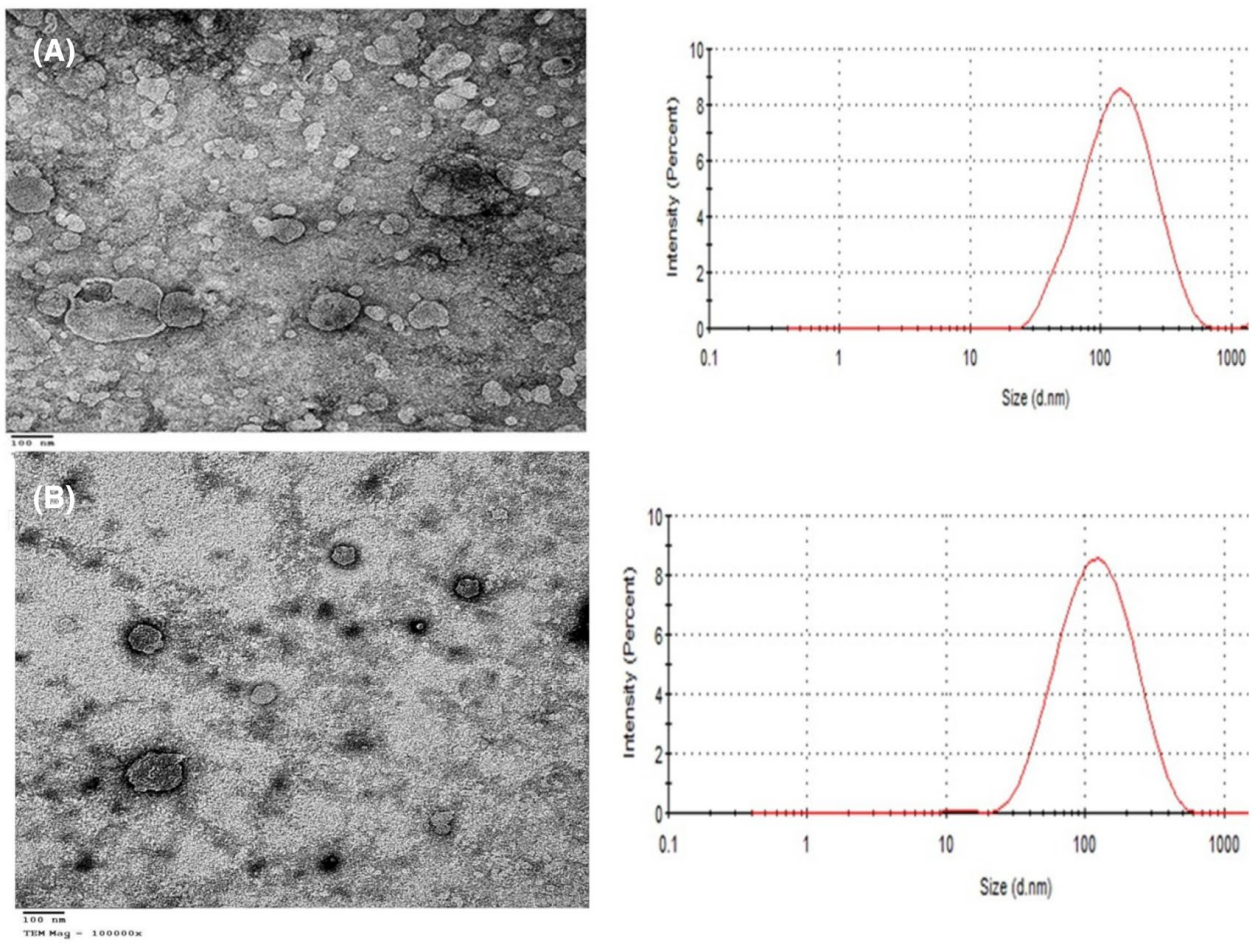
Negative stain electron micrographs (magnification 100000X, scale bar = 100 nm) and particle size distribution curves of (**A**) nobiletin composite PEVs (F13), (**B**) nobiletin composite transfersomes (F17).

Formulations F13 and F17 were selected for examination by the transmission electron microscope. As evident in Fig. 2, the vesicles exhibited spherical, non-aggregated appearance with narrow size distribution similar to those obtained by other authors^9,37^. Worthy to note that the particle size obtained using the TEM imaging was concurring with the particle size results obtained by the zetasizer.

#### Effect of storage on the selected nobiletin vesicular systems

The effect of storage on the properties of formulae (F13 & 17) is demonstrated in supplementary 4. Both formulations displayed good storage properties demonstrated by insignificant changes (*P* > 0.05) in particle size, PDI, zeta potential as well as entrapment efficiency (EE%), which may be ascribed to the presence of chitosan coat on both vesicles. Chitosan has been studied for years as a promising coating material to overcome the instability of liposomes by preventing particles aggregation as well as drug leakage^95^.

#### *In-vitro* cytotoxicity assay of the selected nobiletin vesicles

**Figure 3:**
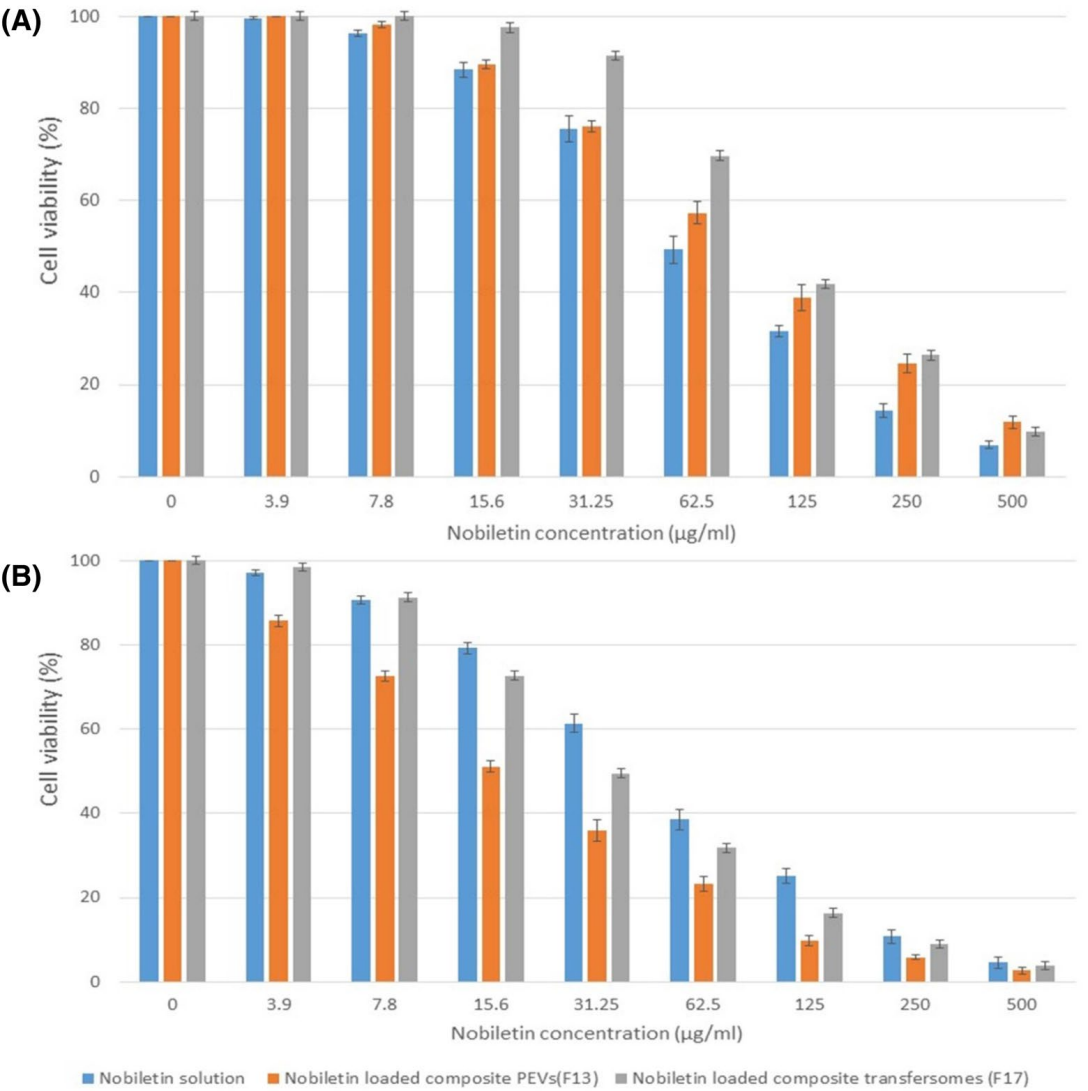
(**A**) Concentration-dependent reduction of cell viability by nobiletin solution and nobiletin vesicular systems after 24 h, calculated for human epidermoid carcinoma A431 cell line using the MTT assay (n = 3), (**B**) Concentration-dependent reduction of cell viability by nobiletin solution and nobiletin vesicular systems after 48 h, calculated for human epidermoid carcinoma A431 cell line using the MTT assay (n = 3).

As evident in Fig. 3, both nobiletin and its vesicular formulations inhibited the cell proliferation/viability of A431 cell line in concentration- and time-dependent manners. After 24 h incubation, the nobiletin solution displayed significantly higher cytotoxicity than formulations F13 and F17 (*P* < 0.05). However, after 48 h incubation the opposite occurred, and the vesicular formulations exhibited significantly higher cytotoxicity than the corresponding nobiletin solution (P < 0.05). These results may be ascribed to the controlled release nature of the drug from the nanoparticles^96^. Similar observations were also reported by other authors^97^.

As shown in Table 3, after 24 h incubation, nobiletin solution exhibited IC_50_ value of 61.7 ± 2.1 µg/ml, which was significantly reduced to 46.7 ± 1.4 µg/ml after 48 h incubation (*P* < 0.05). Formulations F13 and F17 displayed IC_50_ values of 87.1 ± 3.7 µg/ml and 107 ± 7.2 µg/ml respectively (*P* < 0.05) after 24 h, hence delineating that the former exhibited better cytotoxic action, but still both were less cytotoxic than the drug solution. The pattern was reversed after 48 h incubation, and formulations F13 and F17 displayed IC_50_ values of 16.7 ± 0.94 µg/ml and 30.9 ± 1.5 µg/ml respectively (*P* < 0.05), delineating that the vesicular formulations were more cytotoxic than the drug solution, with F13 being again superior to F17. Based on the aforementioned, it can be generally concluded that the encapsulation of nobiletin in composite PEVs and composite transfersomes could preserve the inherent cytotoxicity of nobiletin on human epidermoid cancer A431 cell line, and when incubated for enough time (48 h), it enhances its cytotoxicity owing to the enhanced cellular uptake of the vesicles compared to the drug solution^9^. The cytotoxic activity of nobiletin was proven in several cancer cell lines, which is mainly attributed to its apoptosis-induction potential^98^. Since the nobiletin-loaded composite PEVs formulation (F13) exhibited lower IC_50_ value than the composite transfersomal formulation (F17), it was selected for the in-vivo study.

**Table 3:** IC_50_ values of nobiletin solution, composite PEVs (F13) and composite transfersomes (F17) on A431 cell line after 24 and 48 h (n = 3).

Time (h)	IC_50_ (µg/ml)		
	Nobiletin solution	Nobiletin-loaded composite PEVs (F13)	Nobiletin- loaded composite transfersomes (F17)
24	61.70 ± 2.10	87.10 ± 3.70	107.00 ± 7.20
48	46.70 ± 1.40	16.70 ± 0.94	30.90 ± 1.50

#### *In-vivo* anti-cancer activity of the selected nobiletin vesicles

**Figure 4:**
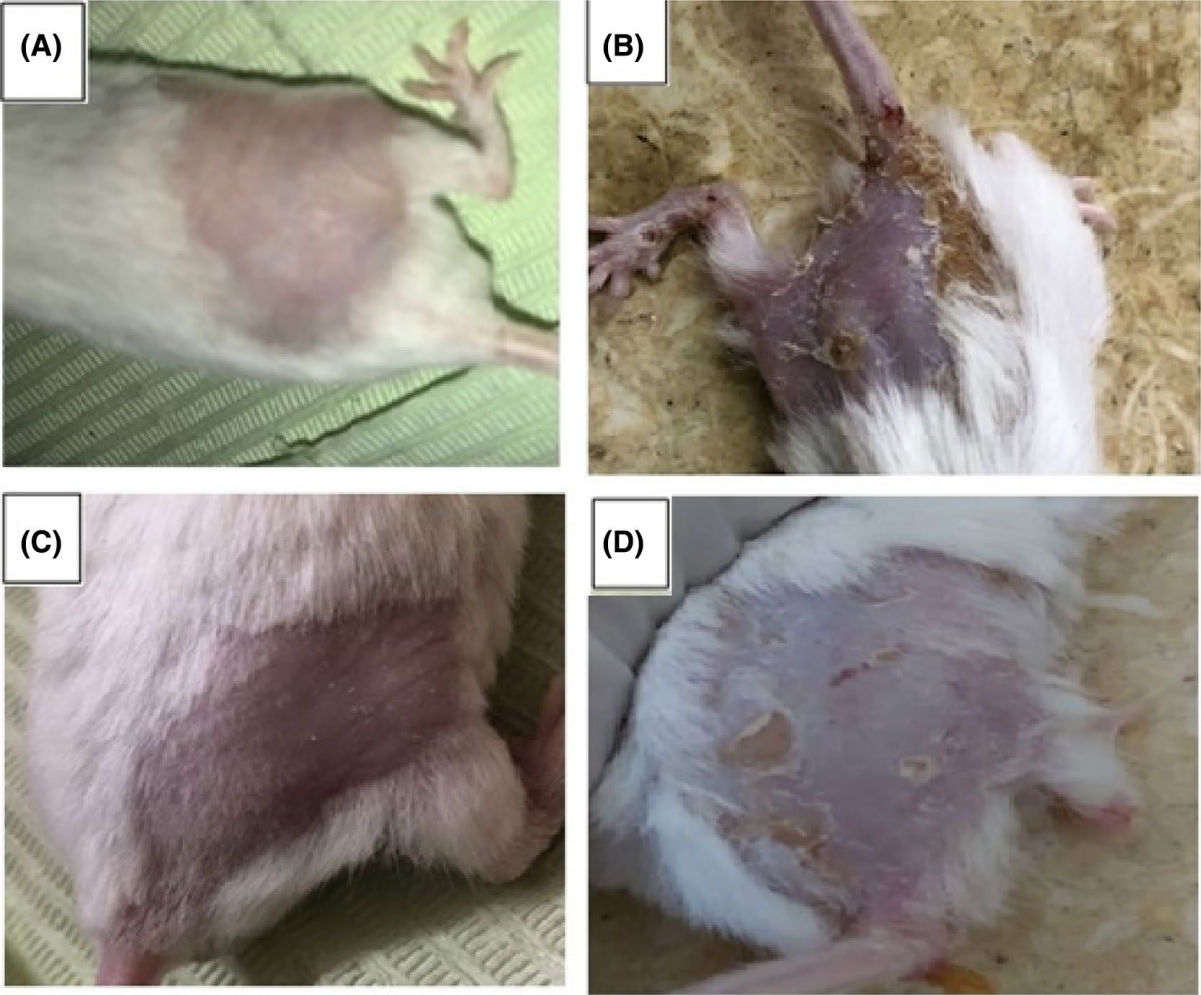
Morphological variations in skin appearance in: (**A**) Negative control group (Group I animals), (**B**) Positive control DMBA-induced skin carcinogenesis group (Group II animals), (**C**) nobiletin composite PEVs formulation (F13) treated group III animals, (**D**) nobiletin solution treated group IV animals.

During tumor induction stage, all carcinogen-treated groups showed general loss of appetite and decrease in activity leading to continuous loss in body weight when compared to the healthy control group (group I) which is caused by the carcinogenicity of DMBA as previously reported by Kaur et al.^99^. The skin morphological difference between groups is shown in Fig. 4. During the treatment period, group III mice treated with nobiletin-loaded composite PEVs formulation (F13) started to regain weight and activity faster than those treated with the nobiletin solution (group IV).

As evident in Fig. 4, the tumor appeared as dispersed patches in the skin, and the daily application of nobiletin composite PEVs (F13) showed an outstanding outcome in treatment of skin cancer, manifested by almost similar skin appearance as the negative control group showed. The application of nobiletin solution also exhibited a treatment ability but to a lesser extent than the vesicular formulation. The in vivo anti- skin cancer activity of nobiletin is ascribed to the inhibition of both nitric oxide and superoxide generation which are closely linked to epithelial carcinogenesis, as well as the suppression of cyclooxygenase 2 and inducible nitric oxide synthase proteins expression, in addition to the inhibition of prostaglandin E2 release^100^. The superiority of the composite PEVs formulation in skin tumor treatment correlates with its superior skin deposition in all skin layers, owing to the nanometric size of the vesicles and the presence of penetration enhancer facilitating dermal delivery of this lipophilic drug^71^. Besides, the presence of chitosan played a key role in enhancing the dermal delivery of nobiletin, attributed to the positive charge of the former which allows it to cause transient opening in the SC, and to adhere to the negatively charged dermal cells by strong electrostatic interaction. Several studies confirmed that the presence of chitosan exhibited enhanced dermal drug penetration.

**Figure 5:**
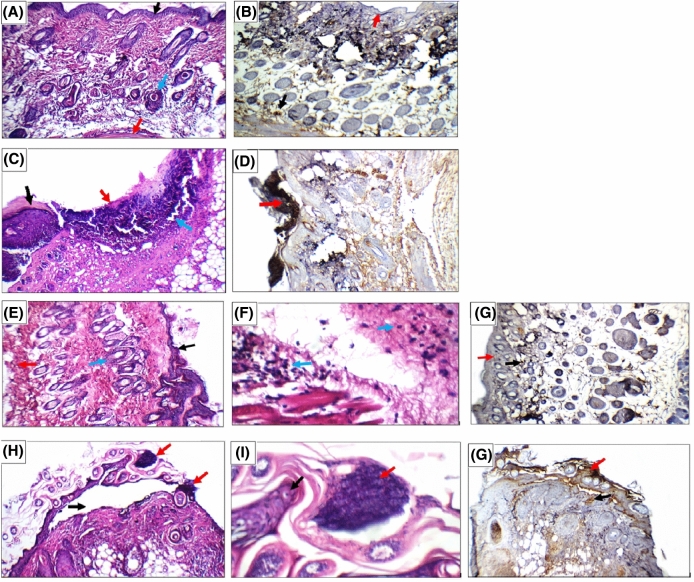
(**A**, **B**) Representing the negative control group skin sections; where (**A**) skin showing average epidermis (black arrow), average dermis with pilo-sebaceous units (blue arrow) and average deep dermis (red arrow) (H&E × 200), (**B**) skin showing positive nuclear reactivity (0) for ki67 in less than 5% in epidermis (red arrow) and in dermis (black arrow) (ki67 immunostain × 200). (**C, D**) Representing the positive control group skin sections; where (**C**) skin showing healthy area (black arrow), and another area of ulceration with infiltrating tumor (red arrow) reaching the deep dermis (blue arrow) (H&E × 200), (**D**) skin showing strong nuclear reactivity (+ +) for ki67 in more than 50% of tumor cells (red arrow) (ki67 immunostain × 200). (**E**, **F**, and **G**) Representing nobiletin composite PEVs (F13) treated group III skin sections; where (**E**) skin showing intact epidermis (black arrow), average superficial dermis with pilo-sebaceous units (blue arrow) and average deep dermis (red arrow) (H&E × 200), (**F**) Another view showing average deep dermis with mild inflammatory infiltrate (blue arrow) (H&E X 400), (**G**) Skin showing positive nuclear reactivity (0) for ki67 in less than 5% in epidermis (red arrow) and in dermis (black arrow) (ki67 immunostain × 200). (**H**, **I**, and **J**) Representing nobiletin solution treated group skin sections;where (**H**) skin showing detached epidermis (black arrow) with overlying tumor infiltrating superficial dermis (red arrow) (H&E × 200), (**I**) high power view showing epidermis (black arrow) with overlying tumor composed of markedly pleomorphic small cells with hyperchromatic nuclei (red arrow) (H&E × 400), (**J**) skin showing positive nuclear reactivity (0) for ki67 in less than 10% in epidermis (red arrow) and dermis (black arrow) (ki67 immunostain × 200).

Histopathological and immunohistochemical examination of the mice skin was carried out in order to examine the internal morphology of the tumor tissues, and was evaluated in terms of ki-67 scoring as previously described^59^. The skin microphotographs of the skin sections excised from mice of different groups are shown in Fig. 5. Group I animals (negative control) showed average epidermis, upper dermis with pilo-sebaceous units and superficial blood vessels, and average deep dermis with underlying muscles. Skin showed positive nuclear reactivity (0) for ki67 in less than 5% in epidermis and dermis (Fig. 5A, B). In DMBA-induced skin carcinogenesis animals (Group II), the skin showed healthy areas alternating with areas of ulceration with infiltrating tumor composed of markedly pleomorphic small cells and hyperchromatic nuclei reaching the deep dermis. The skin showed strong nuclear reactivity (+ +) for ki67 in more than 50% of tumor cells (Fig. 5C, D). On studying the anticancer potential of nobiletin-loaded composite PEVs formulation F13 (Group III), the skin showed intact epidermis covered by thick keratin, average superficial epidermis with pilo-sebaceous units and average deep dermis with mild inflammatory infiltrate. Skin showed positive nuclear reactivity (0) for ki67 in less than 5% in tumor cells, epidermis and dermis, which is similar to the negative control group (Fig. 5E–G). Finally, in the nobiletin solution treated group (Group IV), skin showed detached epidermis with overlying tumor composed of markedly pleomorphic small cells with hyperchromatic nuclei infiltrating only the superficial dermis. Skin showed positive nuclear reactivity (+ +) for ki67 in more than 50% of tumor cells of the detached epidermis, but (0) less than 10% in epidermis and dermis (Fig. 5H–J), suggesting only a slight improvement compared to the positive control group.

The results of the histopathological study revealed that DMBA caused severe skin damage in both epidermal and dermal layers. Upon daily topical administration of nobiletin-loaded composite PEVs formulation (F13) as a cancer chemotherapeutic agent, results showed that they treated the degenerated epidermal and dermal layers of the skin. The tumor almost disappeared after the administration of the composite PEVs formulation (F13), infiltrated the superficial dermis after the administration of the nobiletin solution, and infiltrated the deep dermis in the positive control group. This further proves the deep penetration potential of (F13) compared to the nobiletin solution.

DMBA is considered carcinogenic owing to its ability to generate reactive oxygen species (ROS)^101^. GSH, SOD and CAT are the chief defence antioxidants that rapidly neutralize any molecule with free radical generation potential^102^. Hence, the levels of these antioxidants in tissues decrease in case of skin cancer, and need to be restored for proper treatment. GSH is the chief antioxidant in our cells that plays a pivotal role in the removal of carcinogens by scavenging free radicals. It is a thiol-containing tripeptide, synthesized from glutamic acid, cysteine and glycine, which protects the body from oxidative stress^103^. SOD is a metalloprotein that catalyzes the conversion of superoxide radicals to hydrogen peroxide, which is then converted to water and molecular oxygen by CAT, thus prohibiting ROS cascade generation^104^. Accordingly, to investigate the anticancer activity of nobiletin-loaded composite PEVs, the activities of SOD, GSH and CAT in skin tissues were studied, and results are illustrated in Table 4.

**Table 4:** Levels of skin oxidative stress biochemical markers on DMBA-induced skin carcinogenesis in mice (n = 5).

Animal Group	MDA (µmol/mg)	GSH (µmol/mg)	CAT (U/mg)	SOD (U/mg)
I	0.33 ± 0.07	577.00 ± 61.29	223.80 ± 26.96	373.60 ± 59.60
II	3.35 ± 0.69	142.20 ± 31.06	78.04 ± 21.35	98.60 ± 19.42
III	0.65 ± 0.11	562.60 ± 52.03	218.60 ± 17.21	341.40 ± 21.37
IV	1.17 ± 0.11	430.40 ± 37.56	136.00 ± 8.86	154.80 ± 7.98

On investigating GSH levels, the groups that were receiving nobiletin as solution or in composite PEVs (F13) (groups IV, III respectively) displayed significant increase of the GSH levels compared to the positive control group II (*P* < 0.05). Further inspection of the results showed that the group treated with nobiletin composite PEVs formulation F13 (group III) significantly elevated the GSH level compared to the solution form (group IV) (*P* < 0.05), and was even able to restore the GSH level to normal values, as evident from the statistically insignificant difference compared to the normal group I (*P* > 0.05). The exact same pattern was also observed with SOD and CAT, their levels were normalized after being treated with (F13) and that was superior to the nobiletin solution. Lipids are the prominent targets for oxidative stress, and MDA is the main and most frequently studied lipid peroxidation product. This aldehyde is very toxic and its interaction with proteins or DNA is considered mutagenic^105^. Besides, investigating lipid peroxidation is also a reliable indicative marker for oxidative stress^106^. Regarding LPO, it was found that there was a significant elevation in the level of MDA with group II treated with DMBA compared to the normal level of group I, indicating increased oxidative stress (*P* < 0.05). On the other hand, there was a significant decrease in the level of MDA with groups III and IV receiving nobiletin formulation (F13) or solution respectively (*P* < 0.05) compared to group II, with the former being superior than the latter. The strong antioxidant activity of nobiletin manifested by restoring the levels of GSH, SOD, CAT and decreasing the level of MDA was supported by other studies^107–110^.

microRNAs (miRNA) are small non-coding RNAs, not translated into proteins, however, they interact with messenger RNA (mRNA) either by inhibiting its translation or causing its degradation, hence controlling gene expression. There is an increasing evidence that altered miRNAs expression is a valuable diagnostic marker for various diseases including cancers. In cancer, miRNAs are considered as either oncogenes (also called “oncomir”) whose expression increase in cancer targeting tumor suppressor genes, thus endorse tumor growth, or tumor suppressors which are downregulated in cancer and many studies confirmed that restoring their expression may revert malignancy^111^. In our study, two widely studied miRNAs in skin cancer were selected for evaluation of our proposed system; miRNA 21 as an example of oncogenic miRNA and miRNA 29A as an example of tumor suppressor miRNA^111–113^.

**Figure 6:**
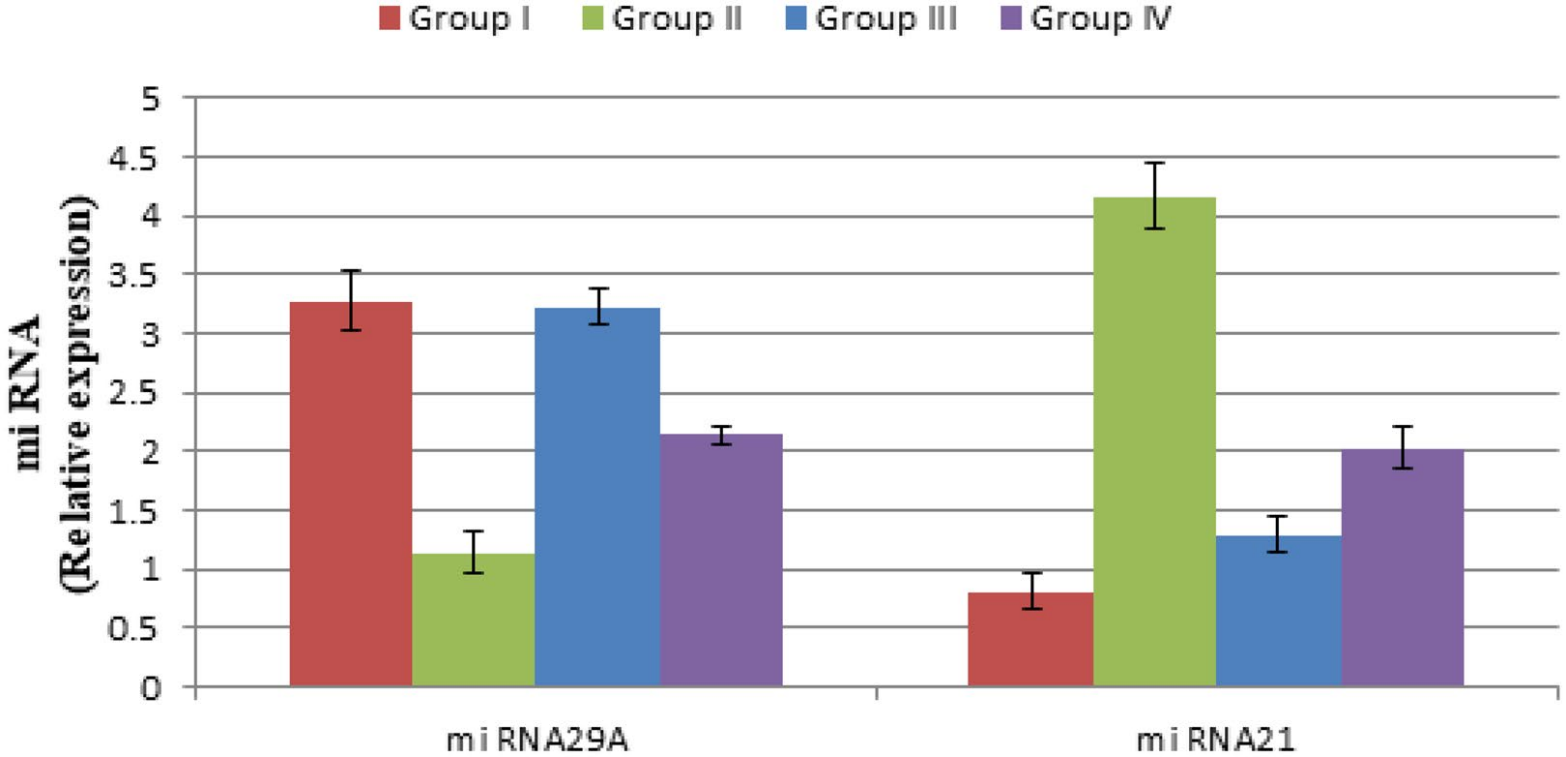
Variations in miRNA29A and miRNA21 expression in skin in DMBA-induced skin carcinogenesis animal model of different groups.

On investigating miRNAs, as evident in Fig. 6, miRNA29A showed significant downregulation in its expression in case of the positive control II compared to the normal group I (*P* < 0.05) which was expected since it is classified as tumor suppressor miRNA. Results showed that nobiletin composite PEVs (F13) restored miRNA29A expression to normal values, as could be depicted from the insignificant difference between groups I and III (*P* > 0.05). Nobiletin solution was also able to significantly elevate miRNA29A level compared to group II (*P* < 0.05), but not like the formulation F13. Regarding miRNA21, which is an oncomir, significant upregulation occurred in its expression in case of the positive control group II compared to the normal group I (*P* < 0.05). Results also confirmed that nobiletin administration significantly decreased miRNA21 expression (*P* < 0.05) compared to the positive control group II, with group III treated with nobiletin composite PEVs formulation F13 being superior to group IV treated with the nobiletin solution (*P* < 0.05). Moreover, the application of the selected formulation F13 did not demonstrate any skin irritation in animals (data not shown), suggesting the tolerability of the formulation. However, despite that the aforementioned formulation displayed satisfactory anti- skin cancer activity, a comparison with a proper topically-applied control such as 5-fluorouracil cream needs to be conducted in a future study, in order to validate the therapeutic efficacy of our proposed formulation.

## Conclusions

Nobiletin vesicular systems were successfully developed and compared for the skin deposition potential. Composite penetration enhancer vesicles composed of phospholipids, transcutol as penetration enhancer, and chitosan demonstrated small particle size, high entrapment for nobiletin with high skin deposition potential. Its promising anti- skin cancer properties were confirmed in vitro on human epidermoid carcinoma cell line A431, and in vivo in which it restored the normal skin condition in DMBA induced skin carcinogenesis mice, as delineated by histological and immuno-histochemical analysis, biochemical assessment of skin oxidative stress biomarkers, in addition to miRNA21 and miRNA29A. Results of this study delineate nobiletin composite penetration enhancer vesicles as a promising candidate for further clinical experimentation.

## Supplementary Information

Supplementary Information.
