# Supplementary material for: Nobiletin-loaded composite penetration enhancer vesicles restore the normal miRNA expression and the chief defence antioxidant levels in skin cancer
Source: Sci Rep. 2021 Oct 12;11:20197. doi: 10.1038/s41598-021-99756-1 (PMC8511031; doi:10.1038/s41598-021-99756-1)
Supplement: Supplementary file 1 — Supplementary Information. [file 41598_2021_99756_MOESM1_ESM.docx]

**Nobiletin-loaded composite penetration enhancer vesicles restore the normal miRNA expression and the chief defence antioxidant levels in skin cancer**

Mahitab Bayoumi^1^, Mona G. Arafa^1,2^, Maha Nasr^3*^, Omaima A. Sammour^3^

^1^Department of Pharmaceutics and Pharmaceutical Technology, Faculty of Pharmacy, The British University in Egypt, Cairo,11837, Egypt.

^2^Chemotherapeutic Unit, Mansoura University Hospitals, Mansoura, 35516, Egypt

^3^Department of Pharmaceutics and Industrial Pharmacy, Faculty of Pharmacy, Ain Shams University, Cairo, 11561, Egypt.

**
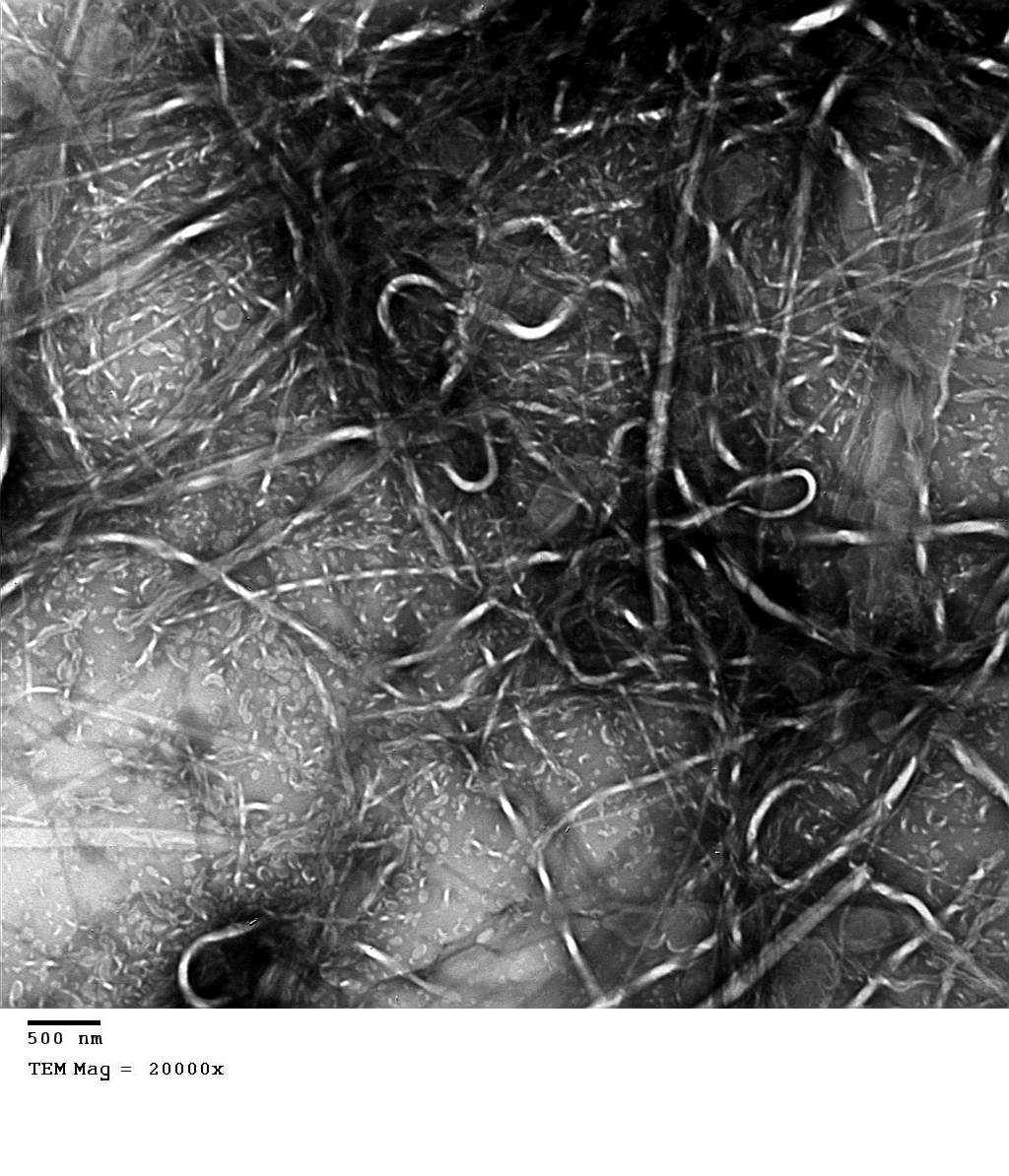
Supplementary 1: TEM picture of a cerosomal formulation displaying tubular morphology**

**Supplementary 2: Viscosity of the selected nobiletin vesicles**

| **Formula**  **code** | **Type of vesicles** | **Viscosity**  **(cp)** |
| --- | --- | --- |
| **F1** | Liposomes | 1.18±0.10 |
| **F5** | PEVs | 1.34±0.16 |
| **F9** | Transfersomes | 1.70±0.12 |
| **F13** | Composite PEVs | 3.64±0.96 |
| **F17** | Composite transfersomes | 4.42±0.10 |
| **F18** | Composite cerosomes | 20.70±2.10 |
| **F22** | Cerosomal PEVs | 21.41±0.60 |
| **F26** | Cerosomal transfersomes | 21.80±1.70 |
| **F30** | Composite cerosomal PEVs | 31.09±1.60 |
| **F34** | Composite cerosomal transfersomes | 39.40±4.30 |

**Supplementary 3: *Ex vivo* skin deposition/permeation data of the selected nobiletin vesicles**

| **Formula code** | **Type of vesicles** | **Nobiletin % permeated in receptor compartment** | **Nobiletin % deposited in S.C.** | **Nobiletin % deposited in epidermis** | **Nobiletin % deposited in dermis** | **Total nobiletin skin deposition %** |
| --- | --- | --- | --- | --- | --- | --- |
| **F1** | Liposomes | 8.20±1.00 | 8.00±0.50 | 27.30±4.50 | 2.70±2.70 | 38.00±10.10 |
| **F5** | PEVs | 20.80±1.10 | 53.70±5.80 | 19.80±1.70 | 4.80±0.20 | 78.30±8.40 |
| **F9** | Transfersomes | 11.30±0.80 | 56.20±0.20 | 26.00±0.70 | 4.50±0.60 | 86.70±2.30 |
| **F13** | Composite PEVs | Nil | 45.80±1.70 | 44.50±0.10 | 5.00±0.20 | 95.30±3.40 |
| **F17** | Composite transfersomes | Nil | 58.30±3.50 | 17.00±1.70 | 24.70±3.50 | 100.00±2.80 |
| **F18** | Composite cerosomes | Nil | 18.00±0.50 | 23.00±3.50 | 13.00±3.60 | 54.00±6.40 |
| **F22** | Cerosomal PEVs | 12.40±0.90 | 15.50±0.70 | 18.10±0.60 | 14.00±0.30 | 47.50±1.30 |
| **F26** | Cerosomal transfersomes | Nil | 17.70±0.10 | 6.00±0.30 | 21.40±0.30 | 45.00±1.20 |
| **F30** | Composite cerosomal PEVs | Nil | 13.20±0.20 | 17.20±0.60 | 2.80±0.20 | 33.20±1.90 |
| **F34** | Composite cerosomal transfersomes | Nil | 19.80±0.60 | 16.30±1.00 | 9.80±0.90 | 46.10±2.30 |

**Supplementary 4: Effect of three months storage at 4±1°C on the particle size, PDI, zeta potential and entrapment efficiency of nobiletin PEVs**

|  | F13 | | F17 | |
| --- | --- | --- | --- | --- |
|  | Before storage | After storage | Before storage | After storage |
| Mean vesicle size (nm) | 126.70 ± 11.80 | 130.30 ± 10.50 | 110.10 ± 0.90 | 113.00 ± 11.30 |
| Mean vesicle PDI | 0.36 ± 0.00 | 0.28 ± 0.10 | 0.39 ± 0.01 | 0.40 ± 0.00 |
| Mean vesicle zeta potential (mV) | +6.10 ± 0.40 | +5.84 ± 0.10 | +9.80 ± 2.60 | +11.70 ± 1.30 |
| Mean vesicle entrapment efficiency | 93.50 ± 3.60 | 90.60 ± 1.78 | 95.60 ± 1.50 | 92.00 ± 2.50 |
